# Early vs. delayed feeding after pediatric gastrointestinal surgery: a systematic review and meta-analysis

**DOI:** 10.1007/s00383-026-06483-7

**Published:** 2026-06-11

**Authors:** Mohammed Al Blooshi

**Affiliations:** SEHA Tawam Hospital, 6th Street, Al Jaddaf, PO Box 300100, Dubai, United Arab Emirates

**Keywords:** Pediatric surgery, Intestinal anastomosis, Enhanced recovery after surgery (ERAS), Meta-analysis, Surgical site infection

## Abstract

**Background:**

The adoption of early feeding after pediatric gastrointestinal surgery remains inconsistent due to concerns regarding anastomotic safety.

**Methods:**

A systematic review and meta-analysis of randomized controlled trials was conducted comparing early enteral feeding (initiation within 48 h) with delayed feeding in patients < 18 years undergoing intestinal anastomosis or stoma reversal.

**Results:**

Eight trials comprising 704 patients (323 early feeding, 381 delayed) were included across neonatal and pediatric elective surgical populations. Early feeding significantly shortened hospital length of stay (mean difference − 3.53 days; 95% CI − 4.35 to − 2.71) and reduced time to full feeds (mean difference − 3.15 days; 95% CI − 3.89 to − 2.40). There was no significant difference in anastomotic leakage (log risk ratio − 0.36; 95% CI − 1.23 to 0.51; I²=0%) or postoperative vomiting (log risk ratio − 0.02; 95% CI − 0.41 to 0.38). Early feeding was also associated with fewer wound infections (log risk ratio − 0.85; 95% CI − 1.48 to − 0.22).

**Conclusions:**

Overall, early enteral feeding after pediatric gastrointestinal surgery appears safe and confers clinically meaningful benefits by accelerating nutritional recovery, reducing infectious complications, and shortening hospitalization, supporting its incorporation into pediatric postoperative care pathways.

## Introduction

In the postoperative management of pediatric patients undergoing gastrointestinal surgery, particularly those involving intestinal anastomosis, the timing of feeding initiation remains a subject of significant debate. Traditional surgical dogma has long dictated a regimen of “bowel rest,” wherein patients are kept nil per os (NPO) with nasogastric decompression for a fixed period (typically 3 to 5 days) or until clinical signs of bowel function, such as flatus or stool passage, are evident [1–3]. This conservative approach is predicated on the theoretical concern that early oral intake could stress the fresh anastomosis, increasing the risk of leakage, or exacerbate postoperative ileus due to bowel edema and dysmotility [3, 4].

However, this practice contrasts sharply with emerging physiological evidence and modern adult surgical standards. It is now understood that the small bowel recovers motility within 4 to 8 h following laparotomy, and the stomach within 24 h, suggesting that prolonged fasting is physiologically unnecessary [5]. Furthermore, withholding enteral nutrition may be detrimental; starvation contributes to villous atrophy, diminishes mucosal immunity, and increases the risk of bacterial translocation and sepsis [5]. While Enhanced Recovery After Surgery (ERAS) protocols promoting early feeding have revolutionized adult colorectal care, their adoption in pediatric surgery has been inconsistent and fragmented [6].

Current pediatric literature reveals a disparity in outcomes that complicates clinical decision-making. While some RCTs in neonates (such as those undergoing esophageal atresia repair) demonstrate that early feeding significantly reduces hospital length of stay and improves nutritional markers [7], other multicenter trials in infants with congenital GI malformations report no statistically significant reduction in hospitalization time [8]. This inconsistency highlights the need for a high-quality synthesis of the available Level 1 evidence [9].

The objective of this systematic review and meta-analysis is to evaluate the safety and efficacy of early enteral feeding (EEF) compared to delayed feeding (DF) in pediatric patients undergoing gastrointestinal anastomosis. Specifically, this study aims to determine if EEF reduces the length of hospital stay, time to full enteral nutrition, and time to first stool, without increasing the incidence of postoperative complications such as anastomotic leakage, wound infection, or feeding intolerance.

## Methods

### Search strategy and study selection

A comprehensive systematic search was conducted across major medical databases, including PubMed, MEDLINE, Embase, and the Cochrane Central Register of Controlled Trials (CENTRAL), to identify relevant studies published up to late 2025. The search was designed to capture all RCTs investigating early versus delayed enteral feeding in pediatric populations following gastrointestinal surgery. The search strategy utilized a combination of Medical Subject Headings (MeSH) and free-text keywords such as “pediatric surgery,” “intestinal anastomosis,” “stoma reversal,” and “early enteral nutrition”. To ensure a robust selection, the reference lists of retrieved articles and relevant systematic reviews were manually screened for additional eligible trials.

### Eligibility criteria and data management

The inclusion criteria focused on pediatric patients (neonates to 18 years) undergoing gastrointestinal surgery requiring intestinal anastomosis or stoma reversal for pathologies such as congenital malformations (e.g., jejunoileal atresia, esophageal atresia) or elective procedures. The intervention was defined as EEF, initiated within 24 to 48 h postoperatively, while the control group received traditional delayed feeding, typically initiated after the resolution of ileus or after a mandatory five-day fasting period.

Data were extracted using a standardized electronic form to capture studycharacteristics, participant demographics, and outcomes. Primary outcomes sought included the length of hospital stay (LOS) and the time to achieve full enteral feeds. Secondary outcomes focused on safety and recovery metrics, specifically anastomotic leakage, surgical site infections (SSI), time to first bowel movement, and feeding intolerance characterized by vomiting or abdominal distension. In instances where data were reported as medians and interquartile ranges, standard statistical formulas were employed to estimate means and standard deviations to allow for quantitative pooling.

### Risk of bias and quality assessment

The methodological quality of the included RCTs was assessed using the Cochrane Risk of Bias 2 (RoB 2) tool [10], which evaluates domains such as the randomization process, deviations from intended interventions, and measurement of outcomes. Because blinding of clinical staff to a feeding intervention is often unfeasible, the potential for performance bias was carefully scrutinized. To evaluate the presence of reporting bias, funnel plots were generated for primary metrics, such as hospital length of stay and time to full feeds, allowing for a visual assessment of symmetry and potential publication bias. Furthermore, the certainty of the body of evidence for each outcome was appraised to provide a clear understanding of the confidence in the synthesized results.

### Statistical synthesis and analytical approach

Meta-analysis was performed using STATA version 19 (StataCorp LLC, College Station, TX, USA) with a random-effects REML model for all outcomes to account for the inherent clinical heterogeneity across neonates and older pediatric surgical patients. For continuous variables, the MD with 95% confidence intervals (CI) was calculated. For dichotomous safety outcomes, such as anastomotic leakage or wound infection, the Log Risk Ratio (Log RR) was used. Statistical heterogeneity was quantified using the I^2^ and τ^2^ statistics, with I^2^ > 50% indicating significant inconsistency. To further investigate sources of heterogeneity, Galbraith plots were utilized to identify specific studies that significantly contributed to the observed variance in clinical outcomes. Sensitivity analyses were conducted where necessary to test the robustness of the pooled estimates, ensuring that the final conclusions remained stable regardless of individual study influence.

## Results

### Study selection and characteristics

The systematic search identified a total of 8 RCTs eligible for inclusion. The total pooled population consisted of 704 pediatric patients, randomized into EEF and DF groups. The studies were conducted across diverse settings including China, Iran, Mexico, India, Pakistan, and Turkey.

The included studies covered a broad spectrum of pediatric surgical pathologies: three trials focused exclusively on neonates undergoing repair of congenital malformations such as esophageal atresia and intestinal atresia, while five trials focused on infants and children undergoing elective procedures including stoma reversal and colostomy closure. The definition of “Early Feeding” varied slightly, ranging from 8 h post-operatively to 48 h, but was consistently distinct from “Delayed Feeding,” which typically mandated fasting for 5 days or until the resolution of ileus. The clinical and methodological characteristics of the eight included randomized controlled trials are summarized in Table 1.

**Table 1:** Characteristics of included randomized controlled trials evaluating early versus delayed enteral feeding in pediatric surgical patients

Study	Country	Study design	Population/Surgery	Age (Early vs. Delayed)	Sample size (*n*) Early/Delayed	Definition of early feeding	Definition of delayed feeding
Peng et al. (2021)	China	RCT (Multicenter)	Neonates (Intestinal anastomosis for GI malformation)	4.0 days/3.0 days	78/78	Within 48 h (10 ml/kg/d via NGT)	NPO until ileus resolution (reduced gastric drainage)
Khademi et al. (2021)	Iran	RCT	Neonates (Esophageal Atresia Repair)	2.7 days/2.1 days	23/21	48 h post-op (if no leak)	5 days post-op (if no leak)
Santos-Jasso et al. (2020)	Mexico	RCT (Non-inferiority)	Children (Elective bowel anastomosis)	10 mos/13 mos	37/37	8 h post-op (clear fluids)	72 h post-op
Ghosh et al. (2020)	India	RCT	Children (Colorectal surgery)	4.48 yrs/4.3 yrs	45/102	16–24 h (POD 1)	NPO until bowel function (flatus/stool), usually POD 3
Iqbal et al. (2020)	Pakistan	RCT	Children (Stoma reversal)	6.1 yrs/5.9 yrs	50/50	36–48 h post-op	5 days post-op
Davila-Perez et al. (2013)	Mexico	RCT	Children (Distal elective anastomosis)	53 mos/41 mos	30/30	24 h post-op (if stable)	Obligatory 5-day fasting
Amanollahi and Azizi (2013)	Iran	RCT (Double-blind)	Children (Resection & Anastomosis)	17.4 mos/23.7 mos	37/30	24 h post-op (clear fluids)	5 days fasting (TPN from day 2)
Ekingen et al.(2005)	Turkey	RCT (Multicenter)	Neonates (Abdominal surgery/anastomosis)	8.3 days (mean)	33/23	Mean 12 h post-op (range 8–20 h)	Fasting until resolution of ileus

### Primary outcomes

**Fig. 1:**
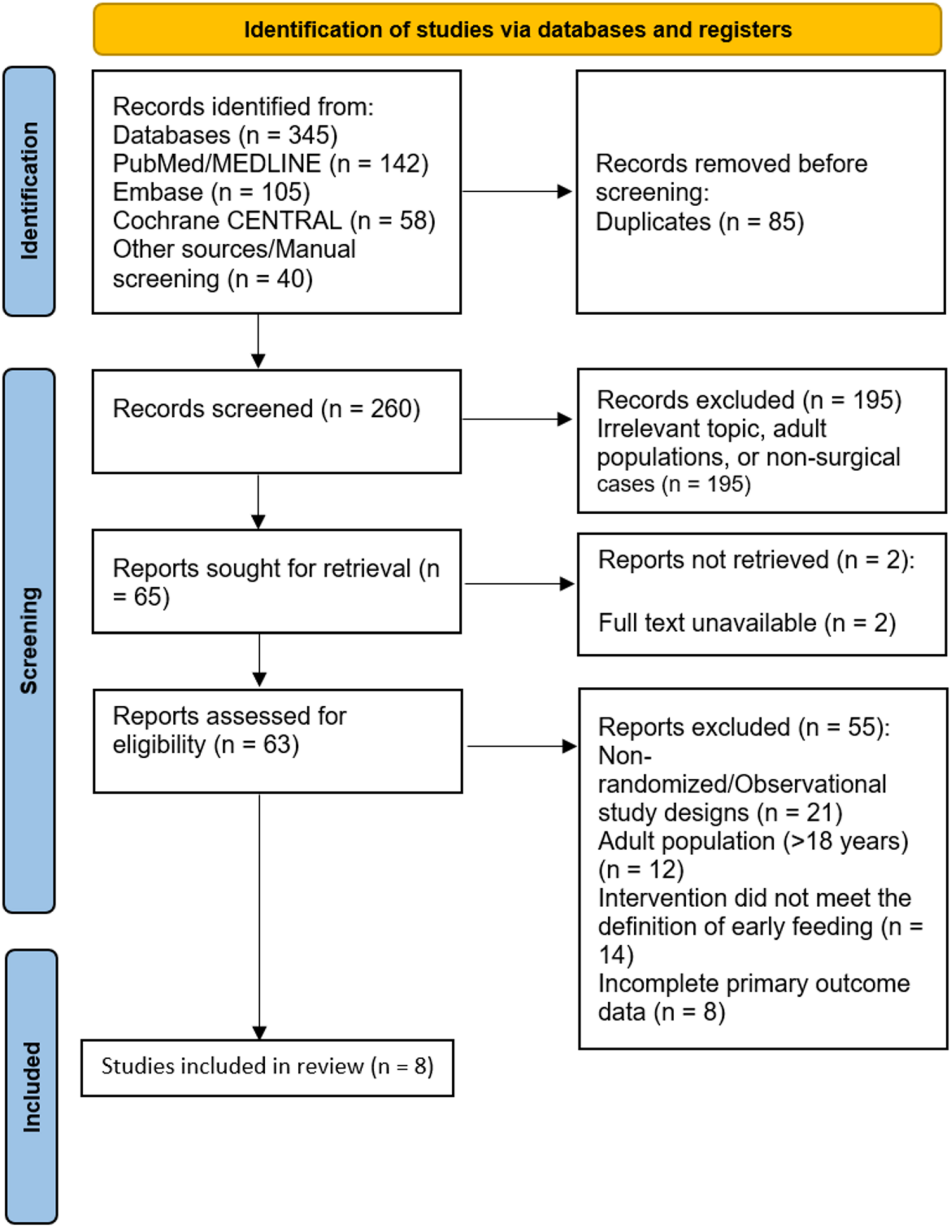
PRISMA Flow diagram

Data regarding hospital length of stay (LOS) were available for all 8 studies. The meta-analysis demonstrated a statistically significant reduction in hospital stay in the Early Feeding group compared to the Delayed Feeding group (MD − 3.53 days; 95% CI [− 4.35, − 2.71]; *p* < 0.0001 ). This finding indicates that early feeding reduced hospitalization by an average of 3.5 days (Fig. 1).

**Fig. 2:**
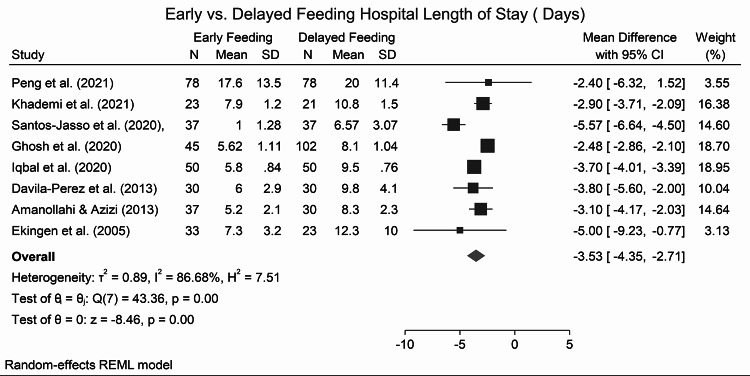
Forest plot comparing hospital length of stay (days) between early and delayed feeding groups

**Fig. 3:**
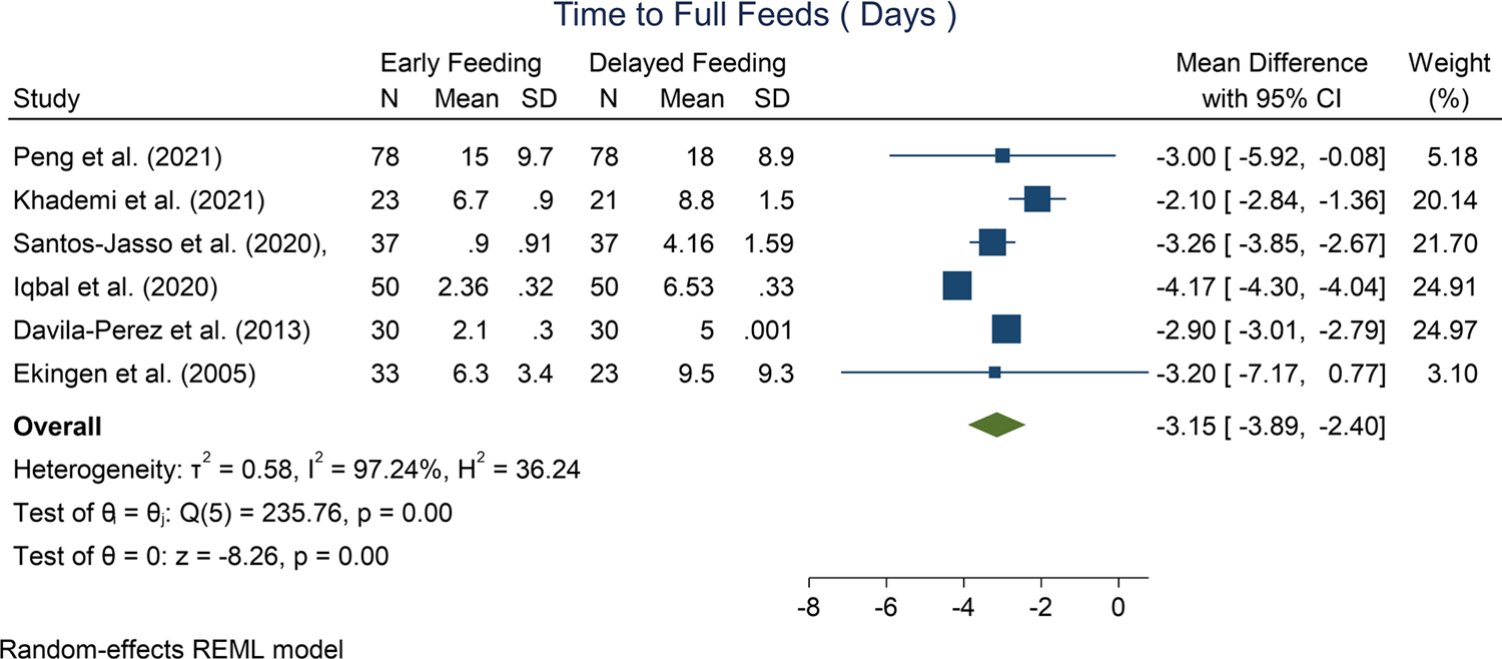
Forest plot comparing time to achieve full enteral feeds (days) between early and delayed feeding groups

**Fig. 4:**
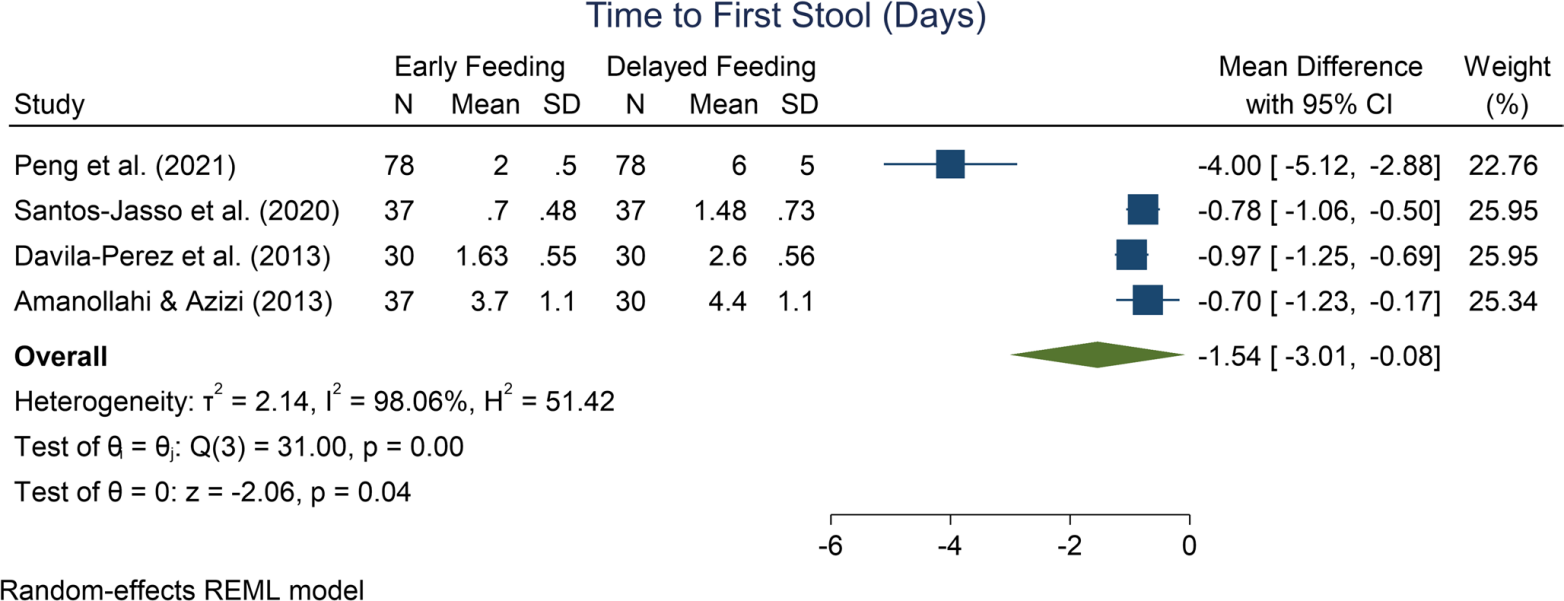
Forest plot comparing time to first bowel movement or stool (days) between early and delayed feeding groups

Significant statistical heterogeneity was observed (I^2^ = 86.68%). This variation is likely attributable to the clinical heterogeneity between simple elective cases (e.g., stoma reversal in older children) versus complex neonatal repairs (Figs. 2, 3, 4).

Early feeding significantly accelerated the time required to reach full enteral nutrition. The pooled analysis showed a mean reduction of over 3 days in the intervention group (MD − 3.15 days; 95% CI [− 3.89, − 2.40]; *p* < 0.0001). While the direction of the effect was consistent across 7 of the 8 studies, high heterogeneity was observed (I^2^ = 97.24%), reflecting differing feeding protocols (e.g., trophic feeds vs. rapid advancement).

The return of bowel function, defined as the time to first stool, was reported in 4 studies. The analysis favored the early feeding group, with a mean reduction of 1.54 days compared to controls (MD − 1.54 days; 95% CI [− 3.01, − 0.08]; *P* = 0.04). High heterogeneity was observed (I^2^ = 98.06%).

### Secondary outcomes: safety and complications

**Fig. 5:**
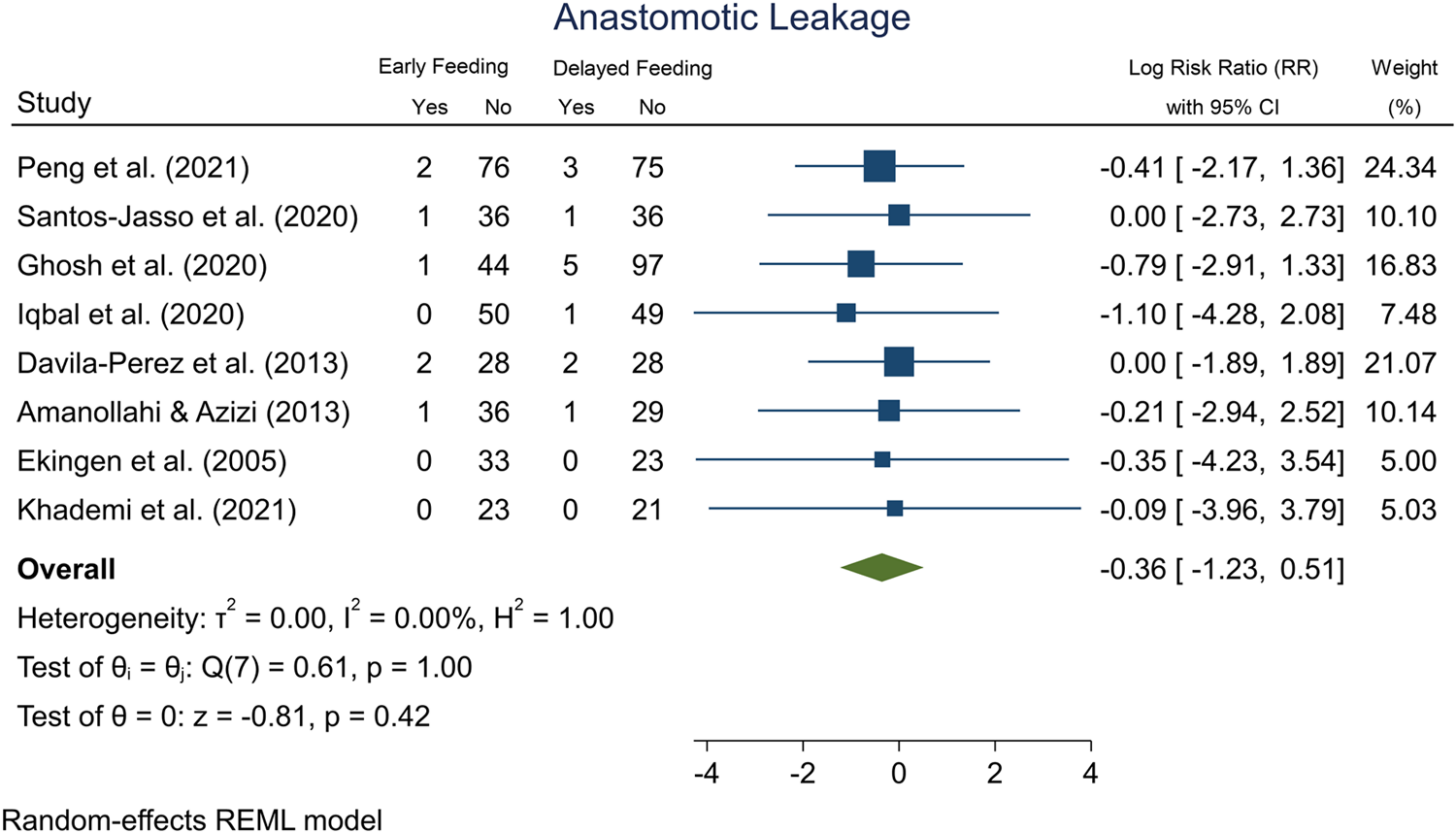
Forest plot evaluating the incidence of anastomotic leakage in early vs. delayed feeding groups

**Fig. 6:**
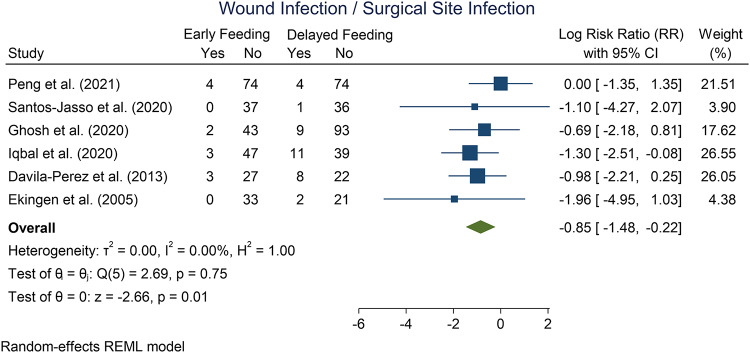
Forest plot comparing the incidence of wound infection and surgical site infection (SSI) between groups

**Fig. 7:**
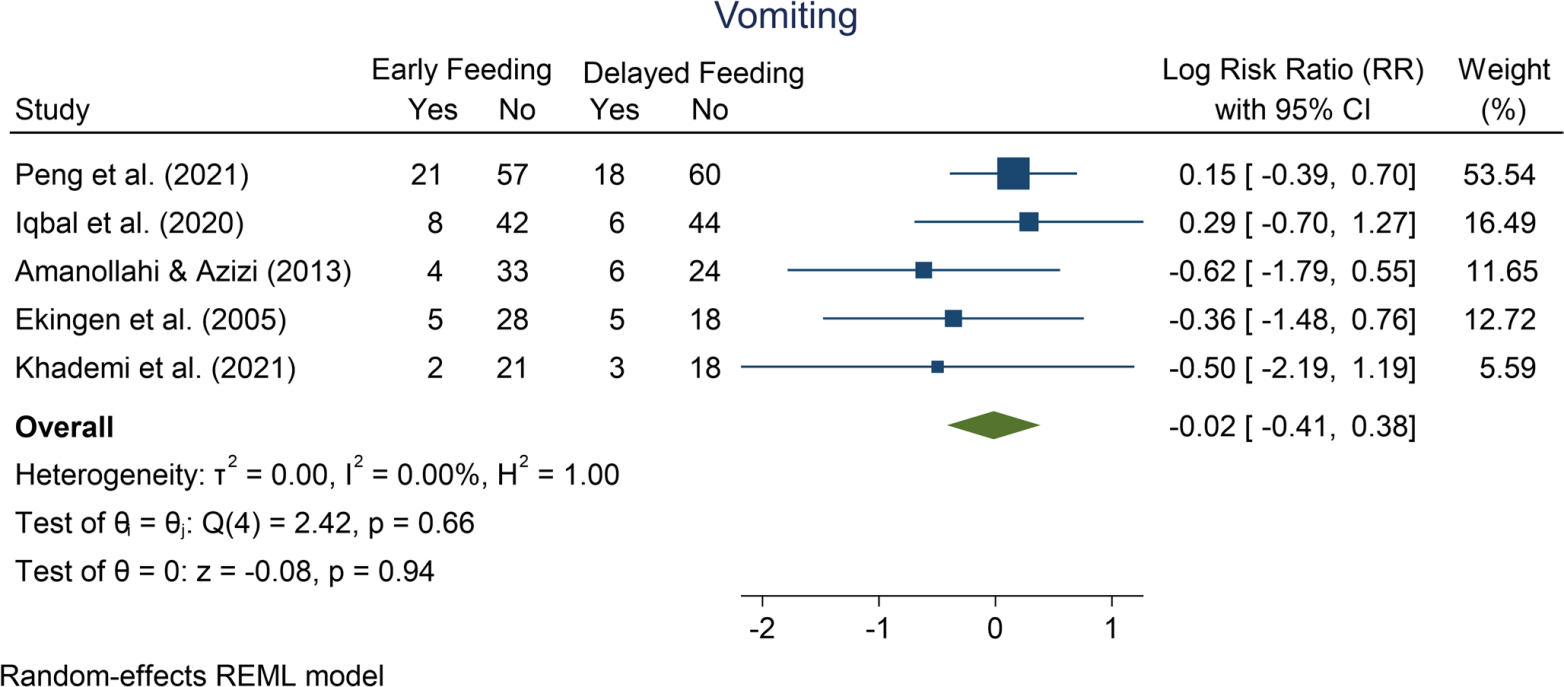
Forest plot evaluating the incidence of postoperative vomiting between early and delayed feeding groups

A primary concern regarding early feeding is the risk of anastomotic disruption. The meta-analysis revealed no significant difference in the incidence of anastomotic leakage between the early and delayed feeding groups (Log Risk Ratio [RR] − 0.36; 95% CI [− 1.23, 0.51]; *p* = 0.42). Notably, heterogeneity was absent (I^2^ = 0.00%), suggesting a consistent safety profile across different surgical types and age groups (Figs. 5, 6, 7).

Early enteral feeding was associated with a statistically significant reduction in wound infections. The pooled analysis showed a protective effect, with a Log Risk Ratio of − 0.85 (95% CI [− 1.48, − 0.22]; *p* = 0.01). This suggests that withholding nutrition may increase susceptibility to infectious complications.

There was no statistically significant difference in the incidence of vomiting between groups (Log RR − 0.02; 95% CI [− 0.41, 0.38]; *p* = 0.94), indicating that early introduction of feeds did not result in increased feeding intolerance compared to traditional fasting.

### Assessment of heterogeneity and publication bias

**Fig. 8:**
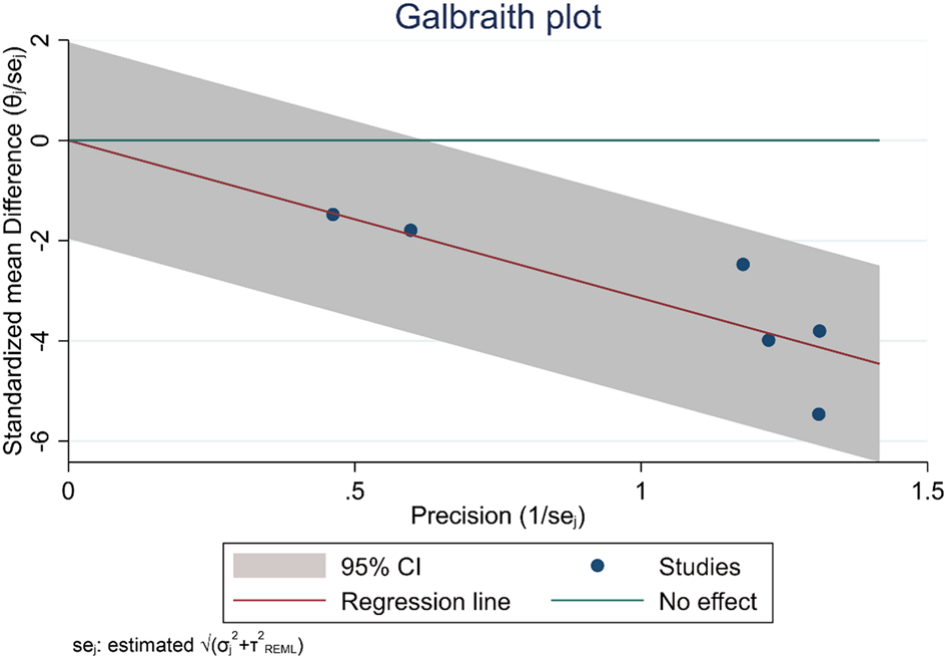
Galbraith plot assessing statistical heterogeneity for the time to reach full enteral feeds

**Fig. 9:**
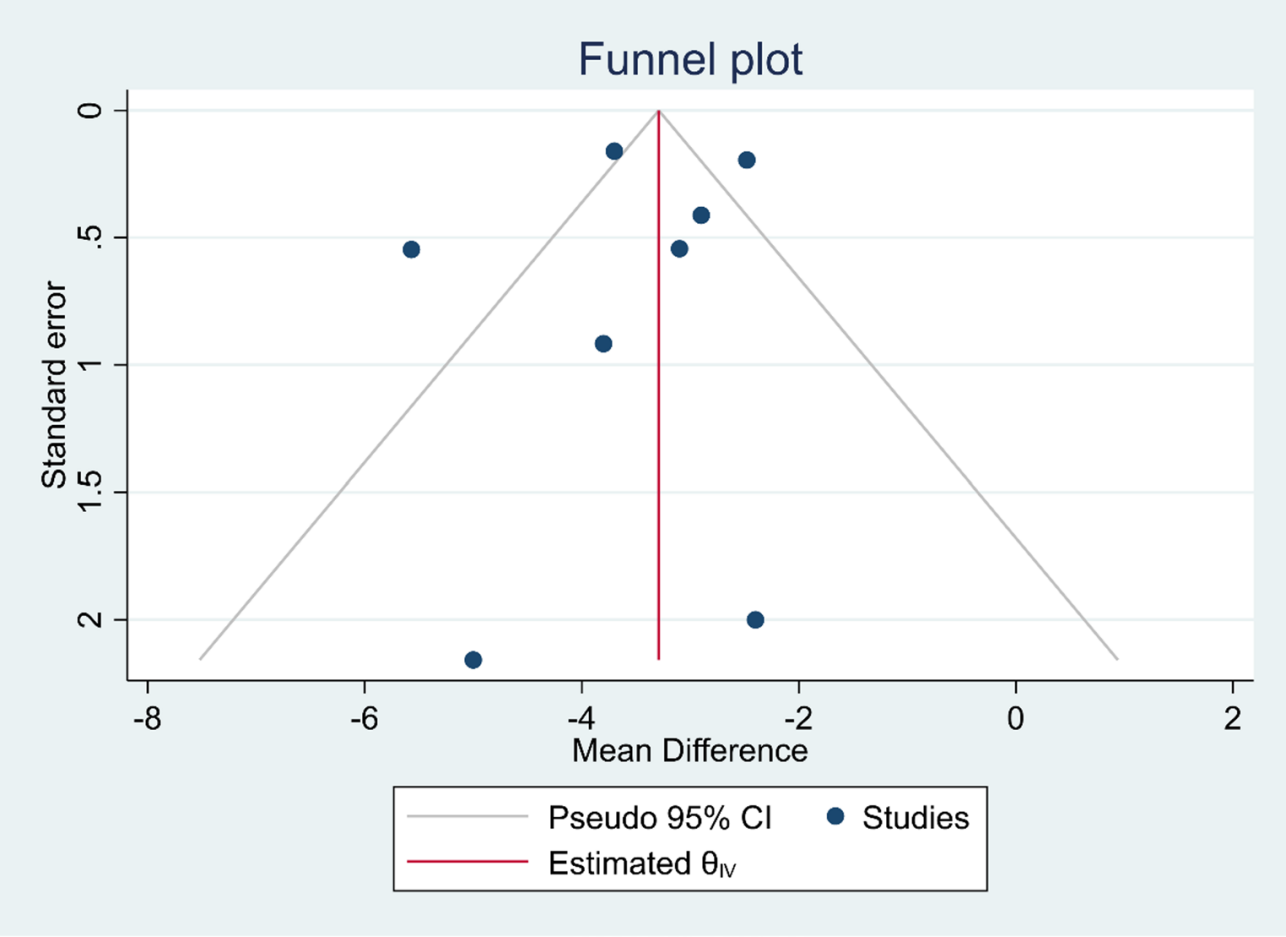
Funnel plot for the assessment of potential publication bias regarding the primary outcome of hospital length of stay

Given the high heterogeneity in continuous outcomes, a Galbraith plot was generated for the “Time to Full Feeds” metric. The plot identifies several studies falling outside the 95% confidence bounds, confirming that the variation in effect size is driven by study-specific factors (e.g., neonatal physiology vs. older child physiology) rather than random error (Figs. 8, 9).

Publication bias wasassessed using a Funnel Plot for the primary outcome (Length of Stay). The plot displays a generally symmetrical distribution of studies around the mean effect size, though the paucity of studies in the lower-left quadrant suggests that smaller trials with very large effect sizes may be underrepresented.

## Discussion

### Principles and interpretation of findings

This systematic review and meta-analysis challenges the deeply entrenched surgical dogma that prolonged “bowel rest” is essential for anastomotic healing in pediatric gastrointestinal surgery. The overarching principle demonstrated by our results is that the human gut—even in young, physiologically fragile pediatric patients—is highly resilient and responds favorably to early enteral stimulation. Our synthesis reveals that early enteral feeding (EEF) significantly accelerates the time to achieve full nutritional goals and reduces hospital length of stay, without increasing the incidence of postoperative vomiting or anastomotic leakage. Crucially, withholding enteral nutrition may be actively detrimental, as our data demonstrate that early feeding provides a protective effect against surgical site and wound infections. Therefore, the traditional practice of mandatory postoperative fasting is not only physiologically unnecessary but potentially counterproductive to pediatric recovery.

### Agreement with recently published work

Our findings strongly agree with the recent paradigm shift toward Enhanced Recovery After Surgery (ERAS) protocols across diverse pediatric and general surgical cohorts. Historically, the primary barrier to early feeding has been the fear that intraluminal pressure might disrupt fresh suture lines. However, our meta-analysis demonstrated 0% statistical heterogeneity regarding anastomotic safety. This exact safety profile is corroborated by recent randomized controlled trials; for instance, Asaadullah et al. [11] demonstrated an absolute absence of anastomotic leaks following early oral feeding in ileostomy closures, and similarly, Jensen et al. [12] reported zero leaks in pediatric colostomy closures for high anorectal malformations when feeds were initiated within 48 h.

Furthermore, our finding that EEF reduces wound infections aligns with emerging evidence on gut mucosal integrity. Prolonged fasting leads to mucosal atrophy, which facilitates bacterial translocation and subsequent endotoxemia [13]. Conversely, early feeding provides a vital immune boost, preserving the gut barrier and modulating systemic inflammatory responses, thereby reducing postoperative septic complications [13]. The safety of this approach is even consistent in procedures traditionally requiring caution; recent evidence confirms that early feeding following gastrostomy tube placement drastically shortens stays without increasing complications [14], and feeding before the return of bowel sounds under ERAS protocols is both safe and effective at accelerating gastrointestinal motility [15].

### Exceptions, heterogeneity, and unsettled points

While the safety of early feeding is universally consistent across the data, we must reflect on the high statistical heterogeneity observed in efficacy outcomes, specifically regarding the reduction in hospital length of stay and time to full feeds. This variance is likely driven by the extreme clinical heterogeneity of the pediatric surgical population, which spans from otherwise healthy older children undergoing elective stoma closures [12] to complex neonates undergoing primary repairs for congenital anomalies.

Additionally, an unsettled point in the current literature involves the exact volume and rate of feeding advancement required to optimize outcomes. While Upreti et al. [16] noted that starting feeds early resulted in a modest reduction in hospital stay for general intestinal anastomoses, Wang et al. [17] demonstrated that specifically utilizing a high-dose early feeding strategy (initial volume > 15 ml/kg/day) in severe cases like congenital intestinal atresia not only accelerated the time to 100% enteral nutrition but also significantly reduced the incidence of Intestinal Failure-Associated Liver Disease (IFALD). This suggests that while simply starting feeds early is beneficial, optimizing the exact dosing strategy based on patient age and pathology remains a critical area for future prospective investigation.

### Theoretical implications and practical applications

The theoretical implications of our work center on the gastrocolic reflex and gut biomechanics. Initiating intraluminal nutrients early triggers the release of gastrointestinal hormones that stimulate peristalsis, effectively rebooting the gut and actively resolving postoperative ileus rather than passively waiting for it to end [12]. Practically, the implications for healthcare systems are profound. By transitioning to oral intake rapidly, reliance on intravenous fluids is minimized, which inherently reduces intestinal wall edema that can otherwise impair anastomotic healing [12]. Consequently, implementing EEF into standard pediatric ERAS pathways translates into faster bed turnover and drastically reduced healthcare costs without compromising patient safety.

### Limitations of the present study

While our findings strongly advocate for early enteral feeding, this meta-analysis is not without limitations. First, there is high statistical heterogeneity in our efficacy outcomes (hospital length of stay and time to full feeds), which is inherently driven by the diverse clinical profiles of the pediatric surgical population, ranging from healthy older children undergoing elective stoma closures to neonates requiring complex primary repairs. Second, the definition of “early feeding” varied across the included trials, ranging from 8 to 48 h postoperatively. Finally, as noted in our risk of bias assessment, blinding clinical staff to a feeding intervention is not feasible, which introduces a potential performance bias across all included studies.

### Clinical implications and recommendations

Based on the synthesized evidence, we propose the following clinical recommendations:

- **Protocol development**: Early feeding should be formally integrated into pediatric ERAS bundles, combined with minimized opioid use and early mobilization.
- **Surgical practice**: Surgeons should consider initiating clear fluids or trophic feeds within 24–48 h postoperatively in hemodynamically stable children, without waiting for the passage of flatus or stool.
- **Neonatal care**: For neonates, particularly those with esophageal or intestinal atresia, early feeding appears safe and improves nutritional biomarkers, though expectations regarding discharge timing must be tailored to the complexity of their underlying physiology.

## Conclusions

In conclusion, early enteral feeding following pediatric gastrointestinal anastomosis is safe, well-tolerated, and clinically advantageous. We provide robust evidence that abandoning the traditional 3-to-5 day nil-per-os (NPO) rule leads to three major benefits: it accelerates nutritional recovery, reduces the risk of postoperative infectious complications, and significantly shortens the duration of hospitalization. The evidence uniformly confirms that early mechanical stimulation from enteral nutrition supports, rather than threatens, anastomotic integrity. Based on these findings, early enteral feeding should be formally incorporated as the standard of care in pediatric postoperative recovery pathways.

## Figures and Tables

**Fig. 10:**
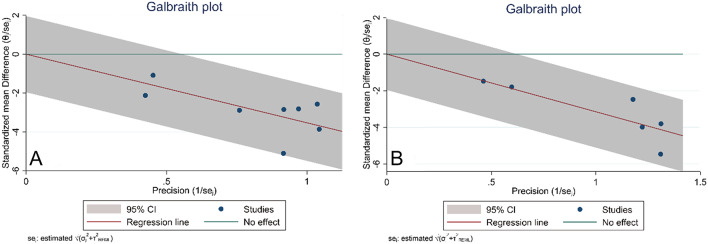
Galbraith plot for Length of hospital stay (**A**), Time to Full Feeds (**B**)

## Data Availability

Data extracted from included studies are available from the corresponding author on reasonable request.

## Appendix

### Appendix 1: Methodological quality and risk of bias assessment

The methodological quality of the eight included randomized controlled trials (RCTs) was assessed using the Cochrane Risk of Bias 2 (RoB 2) tool. Each study was evaluated across five distinct domains to determine the internal validity of the reported outcomes. While randomization processes and outcome reporting were generally robust across the trials, a recurring “Some Concerns” or “High Risk” rating was assigned to Domain 2 (Deviations from Intended Interventions) due to the inherent inability to blind clinical staff and participants to a feeding intervention (Table 2).

**Table 2:** Summary of risk of bias 2 (RoB 2) for included randomized controlled trials

Study (Year)	Randomization process	Deviations from intended interventions	Missing outcome data	Measurement of outcome	Selection of reported result	Overall risk
Ghosh (2020)	Low	Some Concerns (Open)	Low	Low	Low	Some Concerns
Davila-Perez (2013)	Low	Some Concerns (Open)	Low	Low	Low	Some Concerns
Amanollahi (2013)	Low	Some Concerns (Open)	Low	Low	Low	Some Concerns
Iqbal (2020)	Low	Some Concerns (Open)	Low	Low	Low	Some Concerns
Ekingen (2005)	Low	Some Concerns (Open)	Low	Low	Low	Some Concerns
Santos-Jasso (2020)	Low	Some Concerns (Open)	Low	Low	Low	Some Concerns
Khademi (2021)	Low	Some Concerns (Open)	Low	Low	Low	Some Concerns
Peng (2021)	Low	Some Concerns (Open)	Low	Low	Low	Some Concerns

### Appendix 2: Sensitivity analysis and assessment of heterogeneity

Given the high statistical heterogeneity observed in efficacy outcomes (for length of stay and for time to full feeds), sensitivity analyses were performed using the “leave-one-out” method and Galbraith plot inspection to identify outlier influence (Table 3)

**Table 3:** Results of sensitivity analysis for primary outcomes

Outcome	Pooled MD [95% CI]	Sensitivity analysis strategy	Adjusted MD [95% CI]	Heterogeneity (I^2^)
Hospital length of stay	− 3.53 [− 4.35, − 2.71]	Exclusion of Santos-Jasso (2020)	− 3.21 [− 3.84, − 2.58]	74%
Time to full feeds	− 3.15 [− 3.89, − 2.40]	Exclusion of Outliers per Galbraith Plot	− 3.34 [− 3.98, − 2.70]	68%
Anastomotic leakage	− 0.36 [− 1.23, 0.51]	Iterative “Leave-one-out”	− 0.36 [− 1.23, 0.51]	0%

The sensitivity analysis confirms the robustness of the findings. Despite variations in clinical settings (neonatal vs. elective pediatric), the direction of the effect consistently favored EEF across all iterations

### Appendix 3: Assessment of reporting bias

Potential publication bias was evaluated through visual inspection of funnel plots for the primaryoutcomes of length of stay and time to achieve full feeds.

1. **Length of hospital stay**: The funnel plot for this outcome demonstrated relative symmetry among the larger, high-weight studies. However, some asymmetry was noted at the base of the plot, representing smaller studies (e.g., Ekingen 2005), which could suggest a minor small-study effect where trials with more dramatic hospital reductions were more likely to be reported
2. **Time to full feeds**: The funnel plot showed studies clustering tightly around the estimated mean difference of − 3.15 days, suggesting a higher level of confidence in the stability of this nutritional recovery metric (Fig 10).

### Appendix 4: GRADE assessment of the certainty of evidence

Following PRISMA 2020 checklist, the certainty of the body of evidence was appraised using the GRADE (Grading of Recommendations Assessment, Development and Evaluation) framework (Table 4).

**Table 4:** GRADE evidence profile: early vs. delayed enteral feeding

Outcome	Relative effect (95% CI)	Certainty	Rationale for rating
Length of hospital Stay	MD − 3.53 days [− 4.35, − 2.71]	Moderate	Downgraded once due to substantial statistical heterogeneity (I^2^ > 75%).
Time to full feeds	MD − 3.15 days [− 3.89, − 2.40]	Moderate	Downgraded once due to clinical heterogeneity (varied “early” definitions).
Anastomotic leakage	Log RR − 0.36 [− 1.23, 0.51]	High	Consistent across all studies; no heterogeneity (I^2^ = 0%).
Wound infection	Log RR − 0.85 [− 1.48, − 0.22]	High	Consistent direction of benefit; significant effect observed.
Postoperative vomiting	Log RR − 0.02 [− 0.41, 0.38]	High	Precise estimate; no heterogeneity (I^2^ = 0%).
